# Proteome adaptations in Ethe1-deficient mice indicate a role in lipid catabolism and cytoskeleton organization via post-translational protein modifications

**DOI:** 10.1042/BSR20130051

**Published:** 2013-07-25

**Authors:** Tatjana M. Hildebrandt, Ivano Di Meo, Massimo Zeviani, Carlo Viscomi, Hans-Peter Braun

**Affiliations:** *Institut für Pflanzengenetik, Leibniz Universität Hannover, Herrenhäuser Straße 2, 30419 Hannover, Germany; †IRCCS Foundation Neurological Institute ‘C. Besta’, Via Temolo 4, 20126 Milano, Italy; ‡Medical Research Council Mitochondrial Biology Unit, Wellcome Trust/MRC Building, Hills Road, Cambridge CB2 0XY, U.K.

**Keywords:** branched-chain amino acid oxidation, ethylmalonic encephalopathy, hydrogen sulfide, mitochondria, redox regulation, sulfur dioxygenase, BCAA, branched-chain amino acid, BCKDH, branched-chain α-keto acid dehydrogenase, COX, cytochrome *c* oxidase, DTT, dithiothreitol, EE, ethylmalonic encephalopathy, ETF, electron transfer flavoprotein, GAPDH, glyceraldehyde 3-phosphate dehydrogenase, IEF, isoelectric focusing, IPG, immobilized pH gradient, KGDH, 3-oxoglutarate dehydrogenase, PDH, pyruvate dehydrogenase, pI, isoelectric point, PTM, post-translational modification, SCAD, short/branched-chain acyl-CoA dehydrogenase, SDO, sulfur dioxygenase, QPCR, quantitative PCR

## Abstract

Hydrogen sulfide is a physiologically relevant signalling molecule. However, circulating levels of this highly biologically active substance have to be maintained within tightly controlled limits in order to avoid toxic side effects. In patients suffering from EE (ethylmalonic encephalopathy), a block in sulfide oxidation at the level of the SDO (sulfur dioxygenase) ETHE1 leads to severe dysfunctions in microcirculation and cellular energy metabolism. We used an Ethe1-deficient mouse model to investigate the effect of increased sulfide and persulfide concentrations on liver, kidney, muscle and brain proteomes. Major disturbances in post-translational protein modifications indicate that the mitochondrial sulfide oxidation pathway could have a crucial function during sulfide signalling most probably via the regulation of cysteine S-modifications. Our results confirm the involvement of sulfide in redox regulation and cytoskeleton dynamics. In addition, they suggest that sulfide signalling specifically regulates mitochondrial catabolism of FAs (fatty acids) and BCAAs (branched-chain amino acids). These findings are particularly relevant in the context of EE since they may explain major symptoms of the disease.

## INTRODUCTION

ETHE1 is a mitochondrial SDO (sulfur dioxygenase) [1], which takes part in sulfide detoxification by oxidizing persulfides to sulfite [2]. Animal cells produce H_2_S during the catabolism of sulfur-containing amino acids [3], and an additional source is the large intestine where anaerobic bacteria reduce sulfate to sulfide [4]. Endogenous H_2_S functions as a gaseous messenger and has been suggested to be involved in the regulation of several physiological processes such as vascular tone, insulin secretion and inflammation, whereas elevated sulfide concentrations are highly toxic. The exact mechanism of sulfide signalling is largely unknown and thought to be based on the activation of transcription factors as well as direct cysteine S-sulfhydration of target proteins [5,6].

Mutations that lead to a loss of function in ETHE1 disrupt mitochondrial sulfide oxidation and thereby cause the fatal metabolic disorder, EE (ethylmalonic encephalopathy; OMIM #602473) [7,8]. In patients the vascular endothelium is severely damaged by toxic sulfide concentrations in the bloodstream leading to the main symptoms of EE: rapidly progressive neurological failure due to multiple necrotic and haemorrhagic brain lesions, chronic haemorrhagic diarrhoea, vascular petechial purpura and orthostatic acrocyanosis [9]. The biochemical profile, an increase in urinary and plasmatic lactate, acylcarnitine, acylglycine and ethylmalonic acid levels, indicates disturbances in mitochondrial energy metabolism. This can at least partially be explained by sulfide toxicity. H_2_S is a well-known direct inhibitor of COX (cytochrome *c* oxidase) [10], and in addition chronic exposure destabilizes subunits of the enzyme leading to a severe COX deficiency in muscle, brain and colonic mucosa of EE patients [11]. The accumulation of ethylmalonic acid, which is a derivative of butyrate, as well as elevated C4 and C5 acylcarnitines and acylglycines, reflect a block in oxidative metabolism of BCAAs (branched-chain amino acids). Indeed H_2_S has been shown to inhibit one enzyme of this pathway, SCAD (short-chain acyl CoA dehydrogenase), *in vitro* [1].

The severe systemic consequences of a dysfunction in the sulfide oxidation system become obvious in patients suffering from EE. The aim of this study was to identify the effects of elevated sulfide concentrations on the protein composition of different tissues in order to shed light on the role of sulfide in cellular metabolism. For this purpose, the proteomes of liver, kidney, skeletal muscle and brain were analysed in a recombinant Ethe1-deficient mouse model, which faithfully recapitulates the clinical and biochemical features of EE [1].

## EXPERIMENTAL

### Animals

Recombinant Ethe1^−/−^ mice were obtained as described [1]. The mice were maintained on a C57BL6/129Sv mixed background. Four-week-old Ethe1^−/−^ and Ethe1^+/+^ female littermates were considered in this study. Animal studies were in accordance with the Italian Law D.L. 116/1992 and the EU directive 86/609/CEE. Mice were maintained in a temperature- and humidity-controlled animal-care facility, with a 12 h light/dark cycle and free access to water and food (Standard Diet). Mice were killed by dislocation of the neck.

### Protein extraction and 2D gel electrophoresis

Tissue samples were frozen in liquid nitrogen and ground in a shaker mill (Retsch). For IEF (isoelectric focusing) 4 mg of each sample were suspended in 350 μl rehydration buffer (6 M urea, 2 M thiourea, 50 mM DTT (dithiothreitol), 2% CHAPS (w/v), 5% IPG (immobilized pH gradient) buffer 3–11 nl (v/v), 12 μl/ml DeStreak reagent and a trace of bromphenol blue) and homogenized by sonication (3×20 s). Samples were centrifuged at 17000 ***g*** for 10 min and the protein content was adjusted to 2.14 mg/ml (Quantkit, GE Healthcare). IEF was carried out on Immobiline DryStrip gels (18 cm, non-linear gradient pH 3–11) using the Ettan IPGphor 3 system (GE Healthcare). For the second dimension (SDS/PAGE) IPG strips were equilibrated in 6 M urea, 30% glycerol (87%, v/v), 2% SDS, 50 mM Tris/HCl pH 8.8, bromphenol blue with (i) 1% DTT (w/v) and (ii) 2.5% Iodacetamide (w/v) and transferred horizontally onto 16.5% Tricine gels. Electrophoresis was carried out for 20 h at 35 mA/mm gel layer in a Protean IIXL gel system (Biorad) using a broad-range protein molecular mass marker (10–225 kDa, Promega) as molecular mass standard.

### Gel image analysis

Gels were stained overnight with colloidal CBB (Coomassie Brilliant Blue), CBB-250 G (Merck), scanned and analysed with Delta2D software version 4.2 (Decodon). Six gels were used for each tissue (three biological replicates) to compare the protein abundance between Ethe1 knockout mice and wild-type littermates. Only protein spots with significant (Student's *t* test, *P*<0.05), at least 1.5-fold differences in the relative spot volume were assumed to be of different abundance.

### Protein identification by MS

Spots were excised from the 2D gels using a manual spot picker (Genetix), washed with 0.1 M ammonium bicarbonate and dehydrated with acetonitrile. For tryptic digestion the gel pieces were incubated overnight at 37°C (2 μg/ml trypsin in 0.1 M ammonium bicarbonate, Promega). Tryptic peptides were extracted by successive incubation with (i) 50% (v/v) acetonitrile, 5% (v/v) formic acid (ii+iii) 50% (v/v) acetonitrile, 1% (v/v) formic acid (iv) 100% acetonitrile for 15 min at 37°C. Supernatants were pooled and dried by vacuum centrifugation. Peptides were analysed using an EASY-nLC-system (Proxeon) coupled to a MicrOTOF-Q-II mass spectrometer (Bruker Daltonics) according to the protocol described in [12]. Proteins were identified using the MASCOT search algorithm against SwissProt (www.uniprot.org) and NCBI non-redundant protein database (www.ncbi.nlm.nih.gov/protein).

### Data analysis

A co-expression analysis for the Ethe1 gene (Mm.29553, 1417203_at) was performed with Genevestigator (www.genevestigator.com; Nebion). All 2910 wild-type genetic background samples available in the mouse 40 k microarray platform of the manually curated database of Genevestigator were used for the query. Co-expressed genes were identified by using the Pearson correlation coefficient as the measure of similarity. The bioinformatics tool DAVID [13] was used to identify enriched functional annotation terms within the datasets.

### Western blotting

Mouse tissues were homogenized in 10×(v/w)10 mM potassium phosphate buffer, pH 7.4 and centrifuged at 800 ***g*** for 10 min in the presence of protease inhibitors. For the analysis of mitochondrial proteins, an additional high-speed centrifugation step was used to collect the mitochondria. Samples were frozen and thawed twice in liquid nitrogen. Approximately 60 μg of proteins was used for each sample in denaturing SDS/PAGE. Western blot analysis was performed with α-ETHE1 [1], α-ETF (α-electron transfer flavoprotein) [14], α-SCAD (abcam), α-SDH-A (Invitrogen), α-GAPDH (α-glyceraldehyde 3-phosphate dehydrogenase) (Millipore), α-Actin (Millipore) and α-ACTA1 (Dako) antibodies, using the ECL (enhanced chemiluminescence) (Amersham), as described elsewhere [11].

### PCR

Tissue-derived RNA was isolated with TRIzol reagent (Invitrogen). Two micrograms of total RNA was treated with RNase-free DNase and retro-transcribed by using the Cloned AMV First-strand cDNA Synthesis kit and protocol (Invitrogen). Approximately 2–5 ng of cDNA was used for SYBR-GREEN based real-time PCR using primers specific for the amplification of the genes of interest (oligo sequences available on request) according to the ABI-Primer Express software. Standard transcript HPRT (hypoxanthine-guanine phosphoribosyltransferase) was co-amplified using suitable primers. Real-time QPCR (quantitative PCR) was carried out using an ABI PRISM 7000 Sequence Detection System. The amplification profile was according to a two-step protocol: one cycle at 50°C for 2 min, one cycle at 95°C for 10 min and then 40 cycles of 95°C for 15 s and 60°C for 1 min. A final dissociation step (95°C for 15 s, 60°C for 20 s, 95° for 15 s) was added to assess for unspecific primer–dimer amplifications.

## RESULTS

### Effects of Ethe1 deficiency on mouse liver, kidney, brain and muscle proteomes

**Figure 1:**
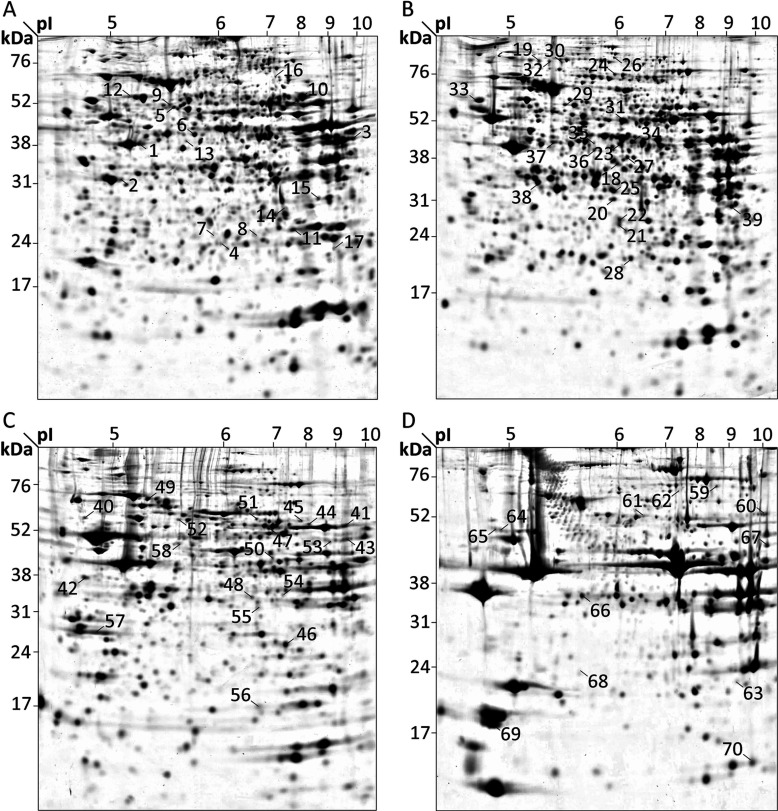
Changes in protein abundance in Ethe1-deficient mouse tissues Total proteins (0.75 mg) from liver (**A**), kidney (**B**), brain (**C**) and muscle (**D**) of three knockout mice and three wild-type littermates were separated by IEF/SDS/PAGE (pH 3–11, 16% acrylamide). The Coomassie Brilliant Blue stained gels were analysed with Delta2D, and significantly different spots (*P*<0.05) with at least 1.5-fold changes in volume are numbered in the fused images. Proteins were identified via nanoLC-MS/MS (see Table 1, Supplementary Table S1).

Total protein extracts from liver, kidney, brain and skeletal muscle were separated by 2D IEF/SDS/PAGE resulting in 70 spots with significantly different volumes in Ethe1-deficient mice compared with wild-type littermates (Figure 1) that contained 81 proteins (Table 1, Supplementary Table S1 available at http://www.bioscirep.org/bsr/033/bsr033e052add.htm). These proteins identified in spots with a changed volume will be described by the shorter term ‘changed/affected proteins’ throughout the paper. In parallel, an unbiased co-expression analysis based on microarray data available in public repositories was carried out using the Genevestigator software. The 81 top scoring genes for co-expression with Ethe1 in wild-type mouse tissues were identified (Supplementary Table S2 available at http://www.bioscirep.org/bsr/033/bsr033e052add.htm) and will be called ‘co-expressed genes/proteins’.

**Table 1:** Proteins with potentially changed abundance in liver, kidney, brain or muscle of Ethe1-deficient mice Proteins were separated by IEF/SDS/PAGE (Figure 1) and spots with at least 1.5-fold changes in volume (k.o./wt) were analysed by nanoLC-MS/MS. For additional information see Supplementary Table S1 (available at http://www.bioscirep.org/bsr/033/bsr033e052add.htm).

Spot	Accession	Name	k.o./wt
Liver			
1	P60710	β-Actin 1	0.43
2	Q64374	Regucalcin	0.53
3	Q91Y97	Fructose-bisphosphate aldolase B	0.54
	Q8VCH0	3-ketoacyl-CoA thiolase B	0.54
4	P97823	Acyl-protein thioesterase 1	0.57
5	Q8R086	Sulfite oxidase	0.57
	Q63836	Selenium-binding protein 2	0.57
6	P29758	Ornithine aminotransferase	0.59
7		Intermediate filament protein	0.62
	O08709	Peroxiredoxin 6	0.62
8	Q80W21	Glutathione S-transferase Mu 7	0.62
9	P30416	Peptidyl-prolyl cis-trans isomerase	0.66
10	Q03265	ATP synthase subunit alpha	1.57
11	P10649	GST Mu 1	1.58
12	P63038	HSP 60	1.66
13	P55264	Adenosine kinase	1.68
14	Q8BH95	Enoyl-CoA hydratase	2.15
15	Q9DCW4	Electron transfer flavoprotein	2.62
16	Q9QXF8	Glycine N-methyltransferase	2.85
17	P35700	Peroxiredoxin 1	3.01
Kidney			
18	Q60866	Phosphotriesterase	0.34
19	P28825	Meprin A subunit alpha	0.37
20	Q9JHW2	Omega-amidase NIT2	0.41
21	Q9QUM9	Proteasome subunit alpha	0.42
22	Q9DBJ1	Phosphoglycerate mutase 1	0.42
23	Q9QYR9	Acyl-coenzyme A thioesterase	0.47
24	Q8CAQ8	Mitofilin	0.51
25	Q99J99	3-mercaptopyruvate sulfurtransferase	0.52
26	Q9WU78	PCD 6-interacting protein	0.53
27	Q60866	Phosphotriesterase	0.53
28	P11352	Glutathione peroxidase 1	0.57
29	P27773	Protein disulfide-isomerase A3	0.58
30	P28825	Meprin A	0.58
31	P47738	Aldehyde DH	0.59
32	P28825	Meprin A	0.61
33	P09103	Protein disulfide-isomerase A1	0.61
34	Q9D964	Glycine amidinotransferase	0.66
35	Q9JK42	PDH kinase	1.64
36	Q9DBL1	SCAD	1.64
37	Q62433	Protein NDRG1	1.67
38	Q6NZD1	Sulfotransferase	1.70
39	Q91VR2	ATP synthase subunit gamma	1.74
	Q64669	NAD(P)H DH	1.74
Brain			
40	P09103	Protein disulfide-isomerase A1	0.44
41	Q03265	ATP synthase subunit alpha	0.47
42	P14206	40S ribosomal protein SA	0.56
	Q61937	Nucleophosmin	0.56
43	O55131	Septin-7	0.59
44	Q64332	Synapsin-2b	0.59
	P06745	Glucose-6-phosphate isomerase	0.59
45	P52480	Pyruvate kinase	0.64
46	P17751	Triosephosphate isomerase	1.54
47	Q03265	ATP synthase subunit alpha	1.58
48	Q61792	LIM and SH3 domain protein 1	1.59
49	P38647	Stress-70 protein	1.61
50	P35505	Fumarylacetoacetase	1.65
51	P80317	T-complex protein 1	1.87
52	P27773	Protein disulfide-isomerase A3	1.88
53	Q91YT0	NADH DH flavoprotein 1	1.94
	P10126	Elongation factor 1-alpha 1	1.94
	O55131	Septin-7	1.94
54	P16858	GAPDH	2.08
55	Q60932	VDAC protein 1	2.14
	P00920	Carbonic anhydrase 2	2.14
56	P17742	Peptidyl-prolyl cis-trans isomerase A	2.19
57	P63101	14-3-3 protein ζ/δ	3.06
58	Q9D2G2	2-oxoglutarate DH E2	3.45
Muscle			
59	Q9JKS4	LIM domain-binding protein 3	0.28
60	Q9JIF9	Myotilin	0.34
61	Q9D0F9	Phosphoglucomutase-1	0.41
62	Q62167	RNA helicase DDX3X	0.53
63	P19157	GST P1	0.61
64	Q00898	α-1-antitrypsin 1-5	0.65
	P62737	Actin, aortic smooth muscle	0.65
65	P62737	Actin, aortic smooth muscle	1.50
	P07758	α-1-antitrypsin 1-1	1.50
66	Q9D6R2	Isocitrate DH	1.58
	Q9JHR4	myosin heavy chain IIB	1.58
67	Q99JY0	3-ketoacyl-CoA-thiolase	1.61
68	P14602	HSPβ1	1.61
69	P97457	Myosin regulatory light chain 2	1.67
70	P04117	FA-binding protein	2.25

In order to obtain a general overview on which pathways or functional categories are associated with Ethe1, we carried out enrichment analysis with the software tool DAVID on the two complete data sets generated by either proteomics or co-expression analysis. The majority of differentially abundant proteins were those regulated by PTMs (post-translational modifications, acetylation and phosphorylation) or with specific subcellular localization (cytosolic and mitochondrial) (Table 2). While the latter result was somewhat expected, given the mitochondrial localization of Ethe1, the former suggests a role for Ethe1 in PTMs. In particular, proteins regulated by acetylation were five times enriched in our sample compared with the whole proteome. Interestingly, similar results were obtained by clustering the genes from co-expression analysis, confirming a close functional connection between Ethe1 and PTMs.

**Table 2:** Functional annotation analysis Proteins with different abundance in Ethe1 deficient compared with wild-type mouse liver, kidney, brain and muscle (proteomics) and of genes that are co-expressed with Ethe1 in wild-type mouse samples (co-expression) were analysed with DAVID.

	Proteomics		Co-expression	
Functional annotation	Number of proteins	Fold enrichment	Number of proteins	Fold enrichment
Acetylation	48	5.0	21	2.0
Phosphorylation	48	1.8	Not enriched	Not enriched
Cytosol	37	2.9	Not enriched	Not enriched
Mitochondrion	30	4.9	38	5.7

**Figure 2:**
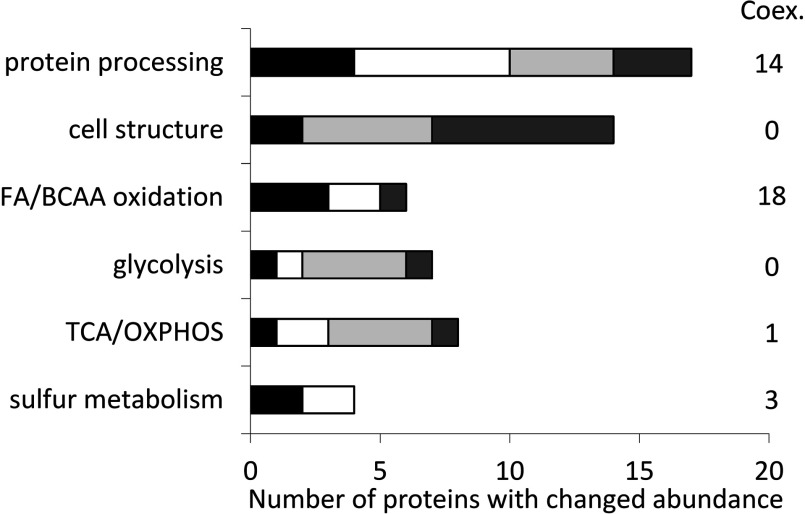
Biological processes affected by Ethe1 deficiency Proteins with different abundance in Ethe1 deficient compared with wild-type mouse liver (black bars), kidney (white bars), brain (light grey bars) or muscle (dark grey bars) were assigned to pathways according to the information provided by UniProt. Coex., number of proteins from the categories listed that are co-expressed with Ethe1 in wild-type mouse samples.

Next, we sorted all proteins into biological processes according to the information available in the Uniprot database (www.uniprot.org) (Figure 2). Among proteins significantly changed, only four were enzymes directly related to sulfur metabolism, namely sulfite oxidase, glycine N-methyltransferase, 3-mercaptopyruvate sulfurtransferase and a sulfotransferase. The largest group of proteins, including 17 enzymes from the proteomic experiment and 14 from co-expression analysis, catalyse reactions during the post-translational processing of proteins, some taking part in general protein handling such as folding (several chaperones, peptidyl-prolyl *cis–trans* isomerase) and degradation (proteasome subunits, proteases), other being involved in specific PTMs (ubiquitinylation, glycosylation, methylation, sialylation, glutathionylation and acylation).

In brain and muscle, we found evidence for an influence on proteins related to cell structure. Actin and myosin were identified in four spots with a changed volume in Ethe1-less mouse muscle. In addition, several proteins that take part in the organization of the cytoskeleton, such as chaperones [T-complex protein 1 and HSPβ1 (heat-shock protein β1)], LIM domain containing or binding proteins, a septin and myotilin, were affected, suggesting that alterations of the cytoskeleton may be involved in the pathogenesis of EE.

Our analysis shows that Ethe1 has a key role in energy metabolism affecting central processes as different as the glycolysis, the TCA (tricarboxylic acid) cycle and the OXPHOS system. Interestingly, the correlation to lipid metabolism was most pronounced. Six enzymes, all catalysing catabolic reactions, were affected by Ethe1 deficiency. Co-expression analysis resulted in 19 genes related to lipid metabolism, including 14 enzymes of FA (fatty acid) or BCAA oxidation, two proteins involved in the regulation of lipid catabolism, two in FA binding and transport and only one enzyme of FA synthesis (Supplementary Table S2). These findings prompted us to investigate further the potential role of Ethe1 in the oxidative catabolism of FAs and BCAAs.

### Effect of Ethe1 deficiency on FA/BCAA catabolic enzymes

**Figure 3:**
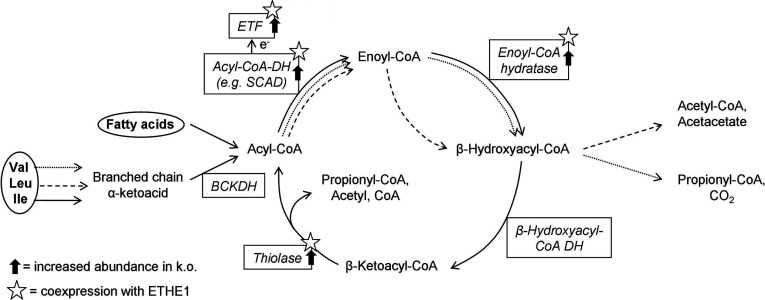
Correlation of Ethe1 and FA/BCAA The enzymes catalysing common reactions in BCAA and FA oxidative metabolism are affected by Ethe1 deficiency (arrows mark increased abundance in Ethe1-deficient mouse liver, kidney, brain or muscle) and also co-expressed with Ethe1 (asterisks). Valine oxidation is shown in solid lines, leucine oxidation in dashed lines and isoleucine oxidation in dotted lines. BCKDH, branched-chain α-keto acid dehydrogenase; SCAD, short/branched-chain acyl-CoA dehydrogenase; ETF, electron transfer flavoprotein.

**Figure 4:**
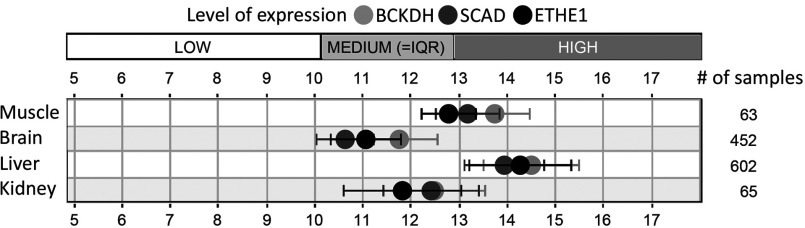
Tissue distribution of Ethe1 and BCAA catabolic enzymes Similar expression pattern of BCKDH (E1 α-subunit), SCAD and ETHE1: gene expression in wild-type mouse skeletal muscle, brain, liver and kidney was analysed using Genevestigator.

**Figure 5:**
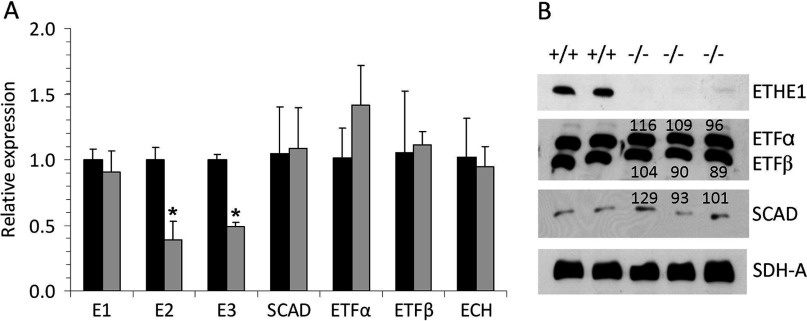
Effect of Ethe1 deficiency on the expression and protein content of enzymes involved in BCAA and FA oxidation in mouse liver (**A**) Gene expression was analysed by QPCR and expression in Ethe^−/−^ samples (grey bars) is shown in relation to the corresponding wild-type samples (black bars). (**B**) Protein abundance was estimated by Western blot. Numbers indicate the relative densities of the bands (percentage of wild-type). E1, E2, E3, subunits of the BCKDH; SCAD, short/branched-chain acyl-CoA dehydrogenase; ETF, electron transfer flavoprotein; ECH, enoyl-CoA hydratase; SDH-A, subunit a of succinate dehydrogenase complex. *Significantly different from wild-type (*P*<0.05).

The BCAAs valine, leucine and isoleucine are oxidized in the mitochondria, and some of the reactions overlap with FA β oxidation (Figure 3) [15]. Enzymes catalysing these common steps were most clearly correlated to Ethe1. As an example, the tissue distribution of Ethe1, SCAD and the committed step in BCAA oxidation, BCKDH (branched-chain keto acid dehydrogenase), on expression level is shown in Figure 4. Four enzymes of FA/BCAA oxidation, SCAD, ETF, enoyl-CoA hydratase and 3-ketoacyl-CoA thiolase, are co-expressed with Ethe1 and also present in spots with an increased volume (1.6- to 2.6-fold) in Ethe1-deficient mice (Figure 3). Separation on the basis of the pI (isoelectric point) detects not only differences in total protein abundance but also some PTMs that modify the charge of the protein. We therefore tested whether these enzymes were actually up-regulated by measuring transcript expression and protein amount (Figure 5). No differences were detected in either expression level or total abundance of SCAD, ETF or enoyl-CoA hydratase indicating an influence on PTMs. In contrast, BCKDH subunits E2 and E3, but not E1, were significantly less expressed in the Ethe1-less mice. Similar to PDH (pyruvate dehydrogenase) and KGDH (3-oxoglutarate dehydrogenase), BCKDH is a large protein complex consisting of three subunits present in multiple copies, subunit E3 being common to all three dehydrogenases. However, the expression of PDH and KGDH E2 subunits was unaffected (results not shown). Notably, these findings clarify the original clinical description of the disease that was thought to be a defect in the β-oxidation pathway.

### Effect of Ethe1 deficiency on PTMs

**Figure 6:**
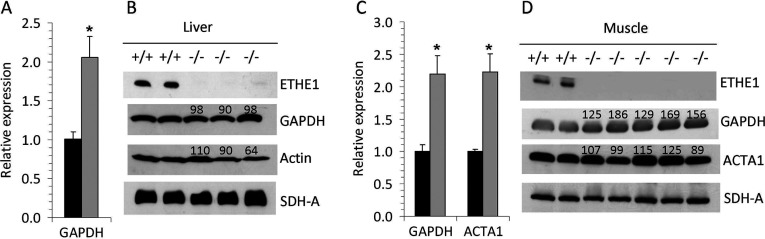
Effect of Ethe1 deficiency on the expression and protein content of GAPDH and actin (**A**, **C**) Gene expression was analysed by QPCR and expression in Ethe1^−/−^ samples (grey bars) is shown in relation to the corresponding wild-type samples (black bars). (**B**, **D**) Protein abundance was estimated by Western blot. Numbers indicate the relative densities of the bands (percentage of wild-type). (**A**, **B**) mouse liver, (**C**, **D**) mouse skeletal muscle. GAPDH, glyceraldehyde-3-phosphate dehydrogenase; ACTA1, actin α1; SDH-A, subunit a of succinate dehydrogenase complex. *Significantly different from wild-type (*P*<0.05).

GAPDH and actin are both known to be regulated by diverse forms of PTMs. Among all proteins affected by Ethe1 deficiency, these two have the highest number of annotations in Uniprot, with GAPDH being subject to ADP-ribosylation, acetylation, methylation, phosphorylation, S-nitrosylation and ubiquitin conjugation, and actin being annotated for acetylation, methylation, nitration, oxidation, phosphorylation and ubiquitin conjugation (Supplementary Table S1). In addition, both proteins are regulated by different forms of cysteine S-modifications such as glutathionylation, palmitoylation and most interestingly sulfhydration, which is not yet included in the annotations [16]. The IEF/SDS results indicate an influence of elevated sulfide and persulfide concentrations on these PTMs. GAPDH was present in a 2.1-fold larger spot in Ethe1-deficient mouse brain that was located at a more acidic pH than the calculated pI of the protein. Such a shift can be caused by different modifications that either introduce an additional negative charge, such as phosphorylation, or mask a positive charge, as in lysine acetylation. A pI shift was also detectable for α actin in muscle with a 1.5-fold decrease in spot 64 and a matching increase in spot 65 at a slightly lower pH (Figure 1). Expression levels of GAPDH and actin were consistently elevated in Ethe1-deficient mice, whereas an increase in total protein abundance was only detectable for muscle GAPDH (Figure 6).

## DISCUSSION

The pathogenic mechanism of EE is related to the accumulation of high levels of hydrogen sulfide in tissues and body fluids of the patients and of the animal model of the disease. While the effects of sulfide on COX have been thoroughly investigated, and shown to be because of an increased degradation of COX subunits [11], the additional systemic consequences are not completely understood.

### Sulfide in protein regulation

Our results suggest that Ethe1 and the sulfide oxidation pathway have a major role in post-translational protein modifications. Proteins that are regulated by such modifications were enriched in both lists of proteins, those affected by Ethe1 deficiency as well as those co-expressed with Ethe1. The data sets also contained many enzymes involved in the catalysis of reactions during the post-translational processing of proteins. Two examples of changed PTM patterns were directly detectable on our 2D gels via acidic spot shifts (actin and septin).

Apart from direct inhibition, cysteine S-sulfhydration is the only known mechanism of enzyme regulation by hydrogen sulfide. Cysteine thiol groups can be converted to hydropersulfides (-SSH), which mostly leads to increased protein activity [16]. Since Ethe1 oxidizes persulfide groups, it could play a role as a negative regulator of S-sulfhydration. The activity as well as stability of enzymes can be regulated by an interaction of different cysteine S-modifications such as S-nitrosylation, S-acylation and S-glutathionylation. We found that acyl-protein thioesterase, which hydrolyses regulatory FAs bound to cysteine thiols, as well as several GSTs (glutathione transferases), which remove cysteine S-glutathionylations, were affected by the knock-out of Ethe1. Thus, our results indicate a general disturbance in the modification of regulatory cysteines, as a consequence of a block in sulfide and persulfide oxidation.

GAPDH is a well-studied example of enzyme regulation by different forms of cysteine S-modifications. It is inactivated by binding of GSH, NO (nitric oxide) or palmitic acid to specific cysteine residues, whereas the activity is 7-fold increased after sulfhydration of Cys^150^ [16–19]. The acidic shift of GAPDH on our 2D gels indicates differences in the PTM pattern. GAPDH expression was increased in liver and muscle of Ethe1-less mice with no concomitant increase in total protein abundance in liver. While in muscle glycolysis is probably up-regulated by the block in mitochondrial respiration, dysregulation might lead to decreased stability of GAPDH in the liver. Similar discrepancies between expression level, total protein abundance and 2D spot size, were also detected for actin and several enzymes of BCAA/FA oxidation, which will be discussed in subsequent paragraphs.

### Sulfide in BCAA/FA catabolism

The metabolite profile in blood and urine of EE patients and Ethe1-deficient mice, characterized by the accumulation of ethylmalonic acid and C4–C5 acylcarnitines, indicates major disturbances in the oxidation of BCAAs and FAs. A similar profile is indeed also present in defects of acyl-CoA dehydrogenases such as SCAD, isobutyryl-CoA dehydrogenase and 2-methylbutyryl-CoA dehydrogenase [20–22]. Thus a simple and elegant explanation for the effect of Ethe1 deficiency on lipid metabolism is the inhibition of these enzymes by accumulating sulfide or persulfides. SCAD is directly inhibited by sulfide [1] and, in addition, CoA persulfide might play a role, as a powerful inhibitor of acyl-CoA dehydrogenases [23,24].

The results presented here reveal an additional level of interaction between Ethe1 and the metabolism of FAs and BCAAs that may explain the original clinical description of the disease as a β-oxidation defect. Lipid catabolism is most clearly co-expressed with Ethe1 and several related protein abundances change as a result of the knock-out of this gene. Interestingly, the prevalence of Ethe1 in liver and muscle parallels the tissue distribution of FA/BCAA oxidizing enzymes [25]. In addition, there is evidence for an influence on the regulatory level. Gene expression for two of the three subunits of the BCKDH complex was decreased in Ethe1^−/−^ liver. As the flux-generating step in the catabolism of BCAAs BCKDH is tightly regulated [26]. In the knockout mice either sulfide or, more likely, the accumulating reaction products of BCKDH down-regulate E2 and E3 subunits. The expression of enzymes catalysing subsequent steps in FA/BCAA oxidation was unchanged so that in this case an effect of sulfide or other metabolites on transcription factors can be excluded. Also sulfide does not interfere with the stability of ETF subunits or SCAD as the block in sulfide oxidation had no influence on total protein abundance. Since direct enzyme inhibition would not be visible on the gels, the differences detected in the 2D proteome are most probably caused by an altered pattern of PTMs. Mitochondrial FA β oxidation has been shown to be regulated by lysine acetylation [27]. Also the enzymes catalysing common steps in FA/BCAA oxidation are subject to different kinds of cysteine S modifications [28,29]. Accumulating sulfide or persulfide probably interferes with this regulatory system. However, the mechanism of this interference will have to be further elucidated in order to fully understand the physiological role of the SDO in lipid metabolism.

A general connection between sulfide and lipid metabolism has previously been reported. Sulfide concentrations were decreased in the plasma of overweight compared with lean men and low sulfide levels are associated with the development of insulin resistance in Type 2 diabetes [30]. Baiges et al. [31] found that Ethe1 and sulfide quinone oxidoreductase, which catalyses the first step in the mitochondrial sulfide oxidation pathway, were decreased by more than 50% in rats fed with a high-fat diet. Thus there seems to be a reciprocal influence of FA and sulfide metabolism with a block of lipid catabolism by high sulfide concentrations and a down-regulation of sulfide metabolism by high concentrations of FAs.

### Sulfide in cell structure

EE patients and Ethe1-deficient mice show severe defects in motor activity and rapidly progressive encephalopathy, both of which are not fully understood yet. Our results show that the increase in sulfide and persulfide concentrations elicits changes in structural proteins as well as in enzymes involved in the organization of the cytoskeleton in brain and muscle, indicating an organ-specific role similar to the isolated COX deficiency, which is also prevalent in these tissues. Accordingly, co-expression analysis provided no hints for a general correlation of genes associated with the cell structure and Ethe1. The reasons for this tissue-specificity are currently unknown. However, it should be mentioned that neither brain–nor muscle–restricted Ethe1 knockout mice, show a clinical phenotype, in spite of tissue-specific COX deficiency, suggesting that the pathogenesis of the disease relies on the absence of Ethe1 in other tissues, particularly in the colon [11]. In fact, the combined administration of NAC (*N*-acetylcysteine), a glutathione precursor and metronidazole, a bactericidal agent against the anaerobic bacteria of the colon, ameliorates the clinical conditions of both mice and patients [32]. Di Meo et al. recently demonstrated that a gene therapy approach based on adeno-associated viral vectors specifically targeting the liver in the very same mouse model used in this study is highly effective in prolonging the lifespan, likely by scavenging most of the circulating sulfide [33].

Our results indicate a role of PTMs in the regulation of cytoskeleton dynamics by sulfide. An intermediate filament protein and actin were present in smaller spots in Ethe1-less mouse liver, but no changes were detected in total actin. Similarly, the actin content of knockout mouse muscle tissue was unchanged although gene expression was increased, and the IEF/SDS results show a higher ratio of modified α-actin. Mustafa et al. identified actin as one of the three most abundant sulfhydrated proteins in sulfide treated mouse liver and estimated that about 12% of the actin molecules are sulfhydrated under basic physiological conditions [16]. Sulfhydration enhances actin polymerization *in vitro* and incubation with 100 μM NaHS leads to rearrangements of the actin cytoskeleton in HEK-293 (human embryonic kidney 293). Sulfhydration itself would not be visible on IEF/SDS gels as the charge of the protein is not changed. However, there could be direct or indirect effects on other forms of modifications such as phosphorylation or acetylation that possibly influence its stability. Sulfide has been shown to induce a reorganization of the actin cytoskeleton in human vascular endothelial cells via the small GTPase Rac1 [34]. Interestingly, we also found a pH shift of the cytoskeletal GTPase Septin 7 in the brain of Ethe1-deficient mice (Figure 1, Table 1, spots 43 and 53) indicating again dysregulation. Additional hints confirming that accumulating sulfide interferes with the organization of the cell structure are changes in abundance of structure specific chaperones and several regulatory cytoskeletal elements. Therefore, the proteomic results show that sulfide signalling must be considered an important factor in the control of cytoskeleton dynamics.

### Additional aspects

Surprisingly, effects of Ethe1 deficiency on sulfur metabolic enzymes were limited. Sulfite oxidase and β-mercaptopyruvate sulfurtransferase were both detected in spots with a decreased volume. A down-regulation of these enzymes would indeed make sense since sulfite concentrations are decreased in the knockout mice and the sulfurtransferase produces additional sulfide. It has been suggested that probably alternative pathways for sulfide detoxification exist at least in liver or kidney, which are currently unknown [1]. However, there is no hint so far about a possible involvement of one of the enzymes found in increased spots in these tissues, glycine-*N*-methyltransferase or sulfotransferase in this process.

H_2_S acts as a cytoprotective agent by activating Nrf2 (nuclear factor-erythroid 2 p45 subunit-related factor 2), which induces the expression of several antioxidant enzymes [6]. In addition, the sulfide molecule itself has antioxidant properties. Our results show that increased sulfide concentrations due to disruption of the sulfide oxidation pathway have several effects on the antioxidant system (e.g. glutathione peroxidase, peroxiredoxin), thus confirming the findings of Palmfeldt et al., who detected alterations in the redox mitochondrial proteome from cultured skin fibroblasts of six EE patients [35].

## Conclusions

Our results provide evidence for a crucial role of the mitochondrial sulfide oxidation pathway in sulfide signalling. Increased sulfide concentrations, due to a block in this pathway at the level of the SDO, lead to major disturbances in post-translational protein modifications, which can explain the pleiotropic devastating effects of EE. This study confirms the involvement of sulfide in redox regulation and cytoskeleton dynamics. In addition, we suggest that sulfide signalling also specifically regulates mitochondrial BCAA/FA catabolism and thus takes part in the coordination of the energy source used by the organism. The mechanism of this regulatory function is most likely based on cysteine S-modifications. Increased sulfide concentrations promote cysteine S-sulfhydrations and thereby shift the balance between different forms of PTMs. In addition, ETHE1 as an SDO could take part in the removal of sulfhydrations by oxidizing the cysteine-derived persulfide group.

## Online data

Table S1 and S2
